# Nasal Catarrh

**Published:** 1880-12

**Authors:** 


HALL'S
### JOURNAL OF HEALTH
AND
MISCELLANY.

E. H. GIBBS, A. M., M. D. - - EDITOR.

Founded in 1854, and published Monthly without Interruption since that Time.

### NASAL CATARRH.

There are two varieties of this complaint. Both are characterized by discharges from the nose. One has no odor ; the other is offensive. The first is only a symptom of cold in the head ; the second is evidence of constitutional trouble. The mild form often seems to be a permanent ailment, owing to the individual's susceptibility to colds, or to his carelessness. Every case can be cured by avoiding colds, keeping the bowels regular, and the feet always dry and warm. Smearing the nose, outside and in, with glycerine, seems to afford great relief. Smelling of spirits of camphor or hartshorn stimulates the nasal passages to throw off the secretions, and, when used promptly and frequently, will sometimes effect a cure in a few hours ; but injecting fluids of any kind into the nostrils, or using snuffs—in this form of catarrh—can only afford temporary relief, and may do permanent injury, by irritating the delicate membrane that lines the passages. There is always the risk of scrofulous persons changing a simple catarrh into the more serious form by meddling too much with it.

There are then two kinds of Nasal Catarrh, and, although they both affect the nasal passages, they are as different in all other respects as any other two diseases. One is a local irritation, with effusion from the mucous surfaces. The tendency, in a healthy person, is always toward recovery, even without treatment.

The other is a scrofulous discharge that has sought an outlet at the point of least resistance.

To cure the catarrh it is necessary to remove the cause. It is probable that some of the advertised "catarrh remedies" may check the discharge. It would be unfortunate if they did, because the disease would almost inevitably be driven to the lungs.

In these severe cases, it is essential to remove the accumulations often, and to keep the passages cleansed and disinfected. In syringing the nose, a "fountain syringe" is best, so that the force of the stream can be easily regulated. All fluids used for this purpose should be as warm as the blood ; never cold.

If the nozzle of a syringe is inserted into one nostril the fluid will flow out at the other, after having passed through both. Always insert the nozzle of the syringe on the side least affected. The fluid will then enter the back portion of the other passage, and force out its contents, so that none can enter the throat and be swallowed. From a pint to a quart of warm water may be used ; and afterward, the patient, lying on his back, should with the little finger put glycerine into the nostrils until he feels it trickle down to the throat ; or he can mix 10 drops of carbolic acid crystals with a teaspoonful of glycerine, and put in a pint of tepid water, and inject the nose with it. For a change, he may use warm soap suds, or a mixture of 10 drops of tincture of iodine, half an ounce of glycerine and one pint of water; or, one drachm of chlorate of potash in one pint of warm water. As before stated, the object is to cleanse and disinfect the parts, and not to dry up the discharge ; and if that should be the result, the remedy that accomplished it ought to be discontinued, unless at the same time the general health has been so much improved that the disease would not be likely to attack the lungs or some other vital part.

Strictly speaking, scrofula is not a disease, but a lack of firmness in the tissues of the body, rendering it susceptible to a certain class of diseases, particularly those affecting the glands. This peculiar condition of the system is termed "scrofulous diathesis." It is generally inherited, but it may be acquired by persons who are naturally healthy, through want and hardships or through disease and intemperance. When the " diathesis " exists, disease meets with less resistance than in the individual who possesses strong tissues and a high degree of vitality.

Nearly all children of delicate frames, and of the precocious or spiritual type, have " scrofulous diathesis," and diseases take firm hold of them. Hence the saying, "the brightest children die first." But good care and good wholesome food—principally meat and milk, abundant exercise and fresh air—always improve and strengthen them, so that in a few years the majority seem to be exempt from what are called scrofulous diseases. Although the system does not respond so promptly to hygienic measures in later life, still the improvement is almost always marked ; and cases of complete recovery from chronic nasal catarrh, by strict attention to the health, are not infrequent.

